# Application of Lipid-Based Nanocarriers for Antitubercular Drug Delivery: A Review

**DOI:** 10.3390/pharmaceutics13122041

**Published:** 2021-11-30

**Authors:** Aristote B. Buya, Bwalya A. Witika, Alain M. Bapolisi, Chiluba Mwila, Grady K. Mukubwa, Patrick B. Memvanga, Pedzisai A. Makoni, Christian I. Nkanga

**Affiliations:** 1 Faculty of Pharmaceutical Sciences, University of Kinshasa, Kinshasa XI B.P. 212, Democratic Republic of the Congo; aristote.buya@unikin.ac.cd (A.B.B.); grady.mukubwa@unikin.ac.cd (G.K.M.); patrick.memvanga@unikin.ac.cd (P.B.M.); 2 Division of Pharmaceutical Sciences, School of Pharmacy, Sefako Makgatho Health Sciences University, Pretoria 0208, South Africa; bwawitss@gmail.com; 3 Department of Pharmacy, Faculty of Pharmaceutical Sciences and Public Health, Official University of Bukavu, Bukavu 570, Democratic Republic of the Congo; alainbapo@gmail.com; 4 School of Health Sciences, Department of Pharmacy, University of Zambia, Lusaka 10101, Zambia; mwilachiluba@gmail.com; 5 Division of Pharmacology, Faculty of Pharmacy, Rhodes University, Makhanda 6140, South Africa

**Keywords:** tuberculosis, antimicrobial resistance, lipid-based nanocarriers, drug delivery, nanomedicines

## Abstract

The antimicrobial drugs currently used for the management of tuberculosis (TB) exhibit poor bioavailability that necessitates prolonged treatment regimens and high dosing frequency to achieve optimal therapeutic outcomes. In addition, these agents cause severe adverse effects, as well as having detrimental interactions with other drugs used in the treatment of comorbid conditions such as HIV/AIDS. The challenges associated with the current TB regimens contribute to low levels of patient adherence and, consequently, the development of multidrug-resistant TB strains. This has led to the urgent need to develop newer drug delivery systems to improve the treatment of TB. Targeted drug delivery systems provide higher drug concentrations at the infection site, thus leading to reduced incidences of adverse effects. Lipid-based nanocarriers have proven to be effective in improving the solubility and bioavailability of antimicrobials whilst decreasing the incidence of adverse effects through targeted delivery. The potential application of lipid-based carriers such as liposomes, niosomes, solid lipid nanoparticles, nanostructured lipid carriers, nano and microemulsions, and self-emulsifying drug delivery systems for the treatment of TB is reviewed herein. The composition of the investigated lipid-based carriers, their characteristics, and their influence on bioavailability, toxicity, and sustained drug delivery are also discussed. Overall, lipid-based systems have shown great promise in anti-TB drug delivery applications. The summary of the reviewed data encourages future efforts to boost the translational development of lipid-based nanocarriers to improve TB therapy.

## 1. Introduction

Tuberculosis (TB) is currently one of the top 10 causes of death worldwide and the deadliest infectious disease, ranking above human immunodeficiency virus and acquired immune deficiency syndrome (HIV/AIDS) [1]. According to the World Health Organisation (WHO), approximately 10 million people were infected with TB in 2020 and 1.5 million died from the disease in the same year. This is equivalent to a staggering 4000 deaths per day [2]. The global distribution of TB cases is not homogenous, and its incidence is highest in developing countries. Approximately 860,000 people have comorbidities of TB and HIV, as well as related syndromes, with 250,000 deaths having been documented in this population [3]. Furthermore, various analyses [4,5,6,7] that have been conducted since the inception of the COVID-19 pandemic have reported that patients with pre-existing TB who become infected with SARS-CoV-2 are at higher risk of developing serious complications from COVID-19, possibly because of the large surface area of the lungs, which makes them highly susceptible to the virus following inhalation [8].

Currently, chemotherapy represents the only hope for the clinical management of patients with TB, achieving cure rates as high as 95% when correctly administered to people with drug-susceptible TB [9]. However, most of the anti-TB drugs exhibit poor pharmacokinetic profiles that often compromise their full potential in clinical settings. Among the problems related to the therapeutic limitations of the existing anti-TB regimens are the poor bioavailability of most anti-TB drugs due to the variable drug absorption and unwanted first-pass metabolism, lengthy regimens with high dosing frequencies, individual and combined drug toxicity, as well as severe adverse effects. These obstacles are partly responsible for the low patient adherence, therapeutic failure, and alarming development of multidrug-resistant strains, thus justifying the current deadly status of TB and the urgent need to improve the existing anti-TB therapy [10,11]. To this effect, considerable attention in TB research has been given to the pulmonary drug delivery strategy, due to the possibility of increasing the drug concentrations in the lungs (i.e., the primary infected tissues) without systemic exposure [12]. For example, Contreras et al. [13] investigated the possibility of the pulmonary delivery of TB drugs, consequently achieving higher drug concentrations in the lungs as well as comparable systemic levels after pulmonary delivery. The authors observed that pulmonary administration of 20 mg/kg rifampicin to guinea pigs exhibited the highest broncho-alveolar drug concentrations and similar systemic drug levels in comparison to the oral and intravenous administrations of 40 mg/kg rifampicin [13].

Another excellent concept embraces the application of particulate systems for the targeted delivery of anti-TB drugs. Because the lungs are naturally endowed with alveolar macrophages, characterized by high phagocytic activity towards any foreign particles, this presents the use of inhalable particulate drug vehicles as a huge opportunity for targeting intramacrophage *Mycobacterium tuberculosis* [14,15]. This was demonstrated by the development of inhaled polylactic acid microparticles co-loaded with isoniazid and rifabutin, showing 20 times higher drug concentrations in infected alveolar macrophages than the pure drugs administered to mice using intratracheal, intravenous, and oral routes [16].

Apart from the intramacrophage delivery concept, colloidal drug carriers have demonstrated the ability to co-encapsulate multiple anti-TB drugs and achieve prolonged drug release in the lungs, with huge potential for reducing the dosing frequency and improving compliance in patients with TB [17]. This was illustrated in a study in which it was observed that alginate-based nanoparticles containing four TB drugs, viz. rifampicin, isoniazid, pyrazinamide, and ethambutol, exhibited high drug accumulation at therapeutic levels for 7–11 days in plasma and for 15 days in the lungs, liver, and spleen, while the free drugs lasted only for 12–24 h [18]. There are many other studies that have reported the potential of nanoparticulate systems to enhance the solubility, stability, bioavailability, and biocompatibility of anti-TB drugs for improved therapeutic outcomes.

The present review summarizes the studies demonstrating the potential of lipid-based nanocarriers in improving the delivery of different anti-TB agents, with an emphasis on their biopharmaceutical attributes. For the purposes of this article, we mainly focus on lipid-based systems commonly investigated for TB drug delivery, including liposomes, niosomes, solid lipid nanoparticles, nanostructured lipid carriers, and emulsions. Manuscripts referenced in the current review were obtained from the Scopus and PubMed electronic databases, using the aforementioned keywords. The titles and abstracts of resultant articles were screened for relevance, following which duplicate and/or irrelevant articles were excluded. Consequently, the review article refers to 129 articles that have been published over 26 years (1995–2021).

We use the term nanoparticles to describe particles with dimensions in the nanometer range.

## 2. Pathogenesis and Management of Tuberculosis

### 2.1. Pathogenesis

TB is a fatal, airborne, chronic, infectious disease caused by *M. tuberculosis complex*, a generic term that includes all the mammalian tubercle bacilli consisting of very similar *Mycobacterium* species [19]. Of these, only *M. tuberculosis* causes human TB, along with *M. canetti*. The other species, such as *M. bovis*, *M. africanum*, *M. microti*, and *M. pinnipedii*, cause TB in domestic and wild animals, alongside the current TB vaccine strain, Bacillus Calmette-Guérin [20,21]. *M. tuberculosis* is mostly characterized by high lipid and mycolic acid contents in the cell wall, an incredibly low rate of division in vitro cell culture, and a naturally high speed of replication in host cells [22,23].

**Figure 1:**
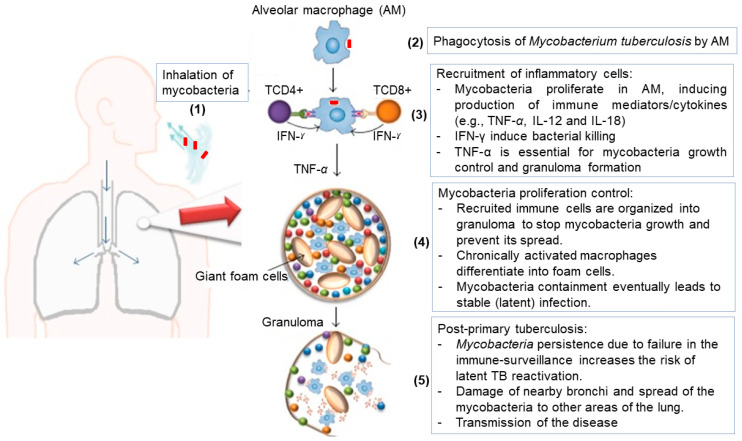
Illustrative description of TB pathogenesis in five consecutive steps: (**1**) mycobacteria entry, (**2**) interactions with alveolar macrophages, (**3**) recruitment and stimulation of immune cells, (**4**) mycobacteria containment in granuloma, (**5**) active TB disease. Adapted from [26].

TB infection is initiated by the inhalation of *M. tuberculosis*, typically carried in aerosol droplets. Once in the deep lung, the tubercle bacillus is taken up by alveolar macrophages through endocytotic mechanisms [24,25]. Alveolar macrophages identify *M. tuberculosis* as a foreign particle and attempt to rid the body of the bacteria via phagocytosis, as depicted in Figure 1. Throughout phagocytosis, alveolar macrophages produce various antimicrobial agents, including proteases, lipases, reactive oxygen and nitrogen species, and a highly acidic environment hostile to the pathogen, as well as proinflammatory cytokines (e.g., Interleukin-6, -12, -18) to recruit other immune cells (i.e., monocytes, neutrophils, and dendritic cells) [26]. In this process, early formed sub-cellular units (named phagosomes) containing the mycobacterium undergo maturation through fusion with lysosomes, which contain the above-mentioned toxic chemicals to form phagolysosomes (mature phagosomes) for extensive digestion of the pathogen. However, the cell wall of *M. tuberculosis* often provides necessary protection that enables the bacillus to resist the immune stress, interfere with the phagolysosomes’ formation, and survive in the cytoplasm of the alveolar macrophages [10,24,27]. In the dormant stage, the mycobacterium resides in the immune cell passively without any replication, leading to an asymptomatic infection commonly known as latent TB infection. In general, only 5–15% of latent cases evolve to active TB disease, with important clinical manifestations such as loss of appetite, chills, fever, night sweats, fatigue, weight loss, and nail clubbing. Nevertheless, the risk for the progression of latent TB infection to active TB disease increases by 10% per year in HIV-positive people and children aged less than 5 years who are household contacts of TB patients. In any case, a diagnosed active TB disease case requires an urgent anti-TB therapeutic regimen to ensure a survival chance of more than 44% for the patient [24,25,28].

### 2.2. Therapeutic Management and Limitations

The treatment of TB relies on a lengthy multidrug therapy that aims to achieve complete cure while preventing transmission, relapse, and the development of drug resistance. The current anti-TB regimen initially involves simultaneous daily administrations of isoniazid (INH), rifampicin (RIF), pyrazinamide (PZA), and ethambutol (ETB) over 2 months. This initial intensive phase is followed by a continuation phase that maintains only INH and RIF for a further 4 months, completing the well-known 6-month regimen [27,28]. In this therapeutic composition, INH combined with ETB (or streptomycin) is expected to provide immediate bacterial clearance towards rapidly replicating tubercular bacilli. The action of PZA is expected to eliminate semi-dormant microorganisms and RIF is intended to supply a slow and permanent sterilizing action on low- or non-replicating bacilli [29]. Although all these antimicrobial agents make a good combination therapy, known as first-line anti-TB drugs, their prolonged frequent administration leads to severe adverse effects that compromise the adherence to the treatment. This often leads to both therapeutic failure and the emergence of new mycobacterium strains that are resistant to one or more of the above-mentioned drugs, leading to drug-resistant or multidrug-resistant TB [10,11]. The emergence of multidrug-resistant strains has led to the establishment of a second line of anti-TB drugs, including various antibiotics, such as ethionamide (prothionamide), kanamycin, amikacin, terizidone, cycloserine, capreomycin, viomycin, and para–aminosalicylic acid, and fluoroquinolones, viz. ofloxacin, levofloxacin, and moxifloxacin. Again, these drugs are almost always given in combinations to slow down the development of resistance; however, the implementation of these therapies has promoted more highly resistant strains to emerge. This continues to cause the rapid broadening of resistant strains that defy concomitant INH (or RIF), a fluoroquinolone analogue, and any other second-line anti-TB drug [30,31,32]. Notably, the complexity of the current TB therapy shows that drug delivery systems that can accommodate multiple drugs are likely to be useful for a very long time to come.

When required for clinical success, the management of multidrug-resistant TB involves the surgical removal of TB lesions in combination with at least four selected therapeutic agents that show good activity against the microbial isolates. The duration of this regimen is extremely long, usually 24 months, which adversely affects patient adherence. Poor patient adherence is often considered to be a result of severe side effects [12,33]. This is particularly challenging for the pediatric populations, where frequent side effects of second-line anti-TB drugs are common and yet there are still insufficient therapeutically related safety data in this patient category. In addition, there is a lack of pediatric dosage forms for these drugs, which exposes children to potential inappropriate dosing, viz. sub-therapeutic or toxic doses [28]. Furthermore, the treatment of HIV-positive patients presents an additional challenge. Significant drug–drug interactions have been observed between the two classes of antimicrobial agents used for TB and HIV. It has been observed that the two therapies may be initiated consecutively. This approach recommends starting primarily with anti-TB agents to render TB tolerant to further dose adjustment, removal, or replacement of some anti-TB drugs prior to starting with anti-retroviral (ARV) therapy. As the problematic component of the TB regimen is one of the most potent anti-TB drugs (RIF), any therapeutic change may raise deep concerns regarding clinical success. The replacement of RIF by rifabutin for HIV–TB coinfection appears to be still limited due to the poor bioavailability and narrowed antimycobacterial spectrum of rifabutin [34]. This shows that while novel therapeutic agents are needed for tackling multidrug-resistant TB strains, it is paramount to find new drug delivery strategies to improve the therapeutic profiles of existing drugs in order to hinder the further emergence of resistant strains [35].

## 3. Lipid-Based Systems as Carriers of Anti-TB Drugs

**Figure 2:**
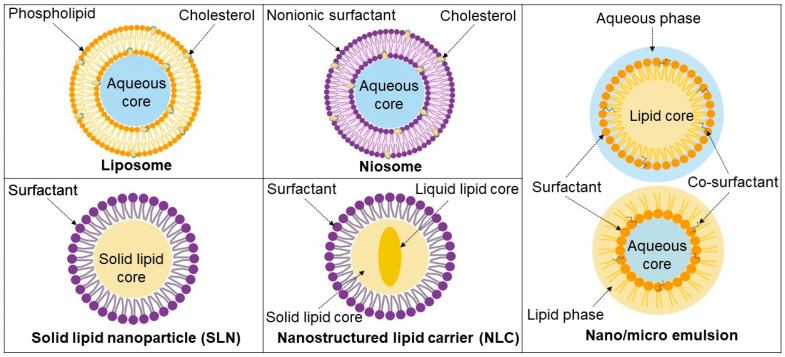
Schematic representation of lipid-based systems discussed herein.

Lipid nanoparticles, as the latest innovation in drug delivery, have considerable potential in the prevention and treatment of infectious diseases including TB. Along with the use of these carriers, it has become possible to improve the apparent solubility and bioavailability of drugs while potentially reducing their toxicity, which are among the main obstacles in the treatment of TB. Lipid-based nanoparticles can be designed as sustained drug delivery systems, thus keeping the drugs in systemic circulation for extended periods. This can significantly reduce the dosing regimen and improve patient adherence. In this section, we provide an overview of some of the most important lipid-based carriers commonly used for the delivery of anti-TB drugs, which are depicted in Figure 2.

### 3.1. Liposomes

Liposomes are the most investigated lipid nanocarriers for the delivery of various medical cargoes, including therapeutic and diagnostic agents. Liposomal particles are spherical vesicles composed of natural or synthetic phospholipids, which are amphiphilic molecules, and cholesterol, which improves in vitro and in vivo stability [36]. Upon aqueous dispersion, phospholipids spontaneously form a bilayer, with the polar head facing the aqueous phase and non-polar tails facing towards each other [37]. The resultant inner aqueous core incorporates hydrophilic molecules, while hydrophobic molecules are encapsulated in the lipid bilayer.

Morphologically, liposomes can be classified as small unilamellar vesicles (SUVs), large unilamellar vesicles (LUVs), giant unilamellar vesicles (GUVs), oligolamellar vesicles (OGVs), and multilamellar vesicles (MLVs) [37,38,39]. Unilamellar vesicles are composed of a single lipid bilayer and can be subcategorized as small when the size is ≤100 nm, while large and giant vesicles are >100 nm but <1 μm, and ≥1 μm, respectively. Oligolamellar vesicles refer to vesicles containing two to five concentric lamellae, whereas multilamellar vesicles have more than five lamellae. These different structures can be distinguished using microscopy imaging. Unilamellar vesicles contain a large aqueous core and are suited for the encapsulation of hydrophilic drugs, whilst multilamelar vesicles are more suitable for lipophilic drugs [37,40].

Liposomes have been widely used as safe and effective drug delivery systems in various diseases, including TB. Their use improves the therapeutic index of anti-TB drugs thanks to their targeting abilities, particularly for pulmonary administration. The integrity of liposomes is severely compromised by the gastro-intestinal and blood environment, resulting in the rapid release of the encapsulated drug. Once administered, traditional liposomes are rapidly cleared from blood circulation by the macrophages’ reticuloendothelial system. Hydrophilic polymers such as polyethylene glycol (PEG) are increasingly used to provide a protective hydrophilic layer on the surface of the liposomes, thereby reducing their elimination from macrophages [41].

### 3.2. Niosomes

Niosomes are similar to liposomes but are composed of non-ionic surfactants that form the bilayer. They are hydrated mixtures of non-ionic surfactants and cholesterol. Commonly used surfactants include polysorbates (Tweens^®^), sorbitan fatty acid esters (Spans^®^), and polyethoxy fatty ethers (Brijs^®^) [42]. As with liposomes, cholesterol is added to increase vesicle stability and bilayer rigidity. The addition of non-ionic surfactants has been reported to increase the particle size of the vesicles, which in turn causes an increase in encapsulation efficiency [43]. Hydrophilic drugs are incorporated into the aqueous core, whereas lipophilic compounds are encapsulated into the lipophilic bilayers. Niosomes are very similar to liposomes in terms of functionality in that they are capable of increasing drug solubility and bioavailability. However, they alleviate the issues associated with conventional liposomes, such as chemical stability and high cost [44,45]. In addition, they have higher penetration potential than conventional liposomes [46,47,48].

### 3.3. Solid Lipid Nanoparticles

Solid lipid nanoparticles (SLN) are colloidal carriers made of biodegradable lipids that are solid at room and body temperature [49]. The lipids used in synthesizing SLN include long-chain triglycerides or partial glycerides, fatty acids, phospholipids, and waxes [50]. Depending on the lipid core, it is usually required to add a stabilizer such as a surfactant (e.g., polysorbate 80, poloxamer 188, lecithin) with a concentration ranging between 1 and 5% (*w*/*v*) [51]. Bioactive compounds, especially lipophilic molecules, are encapsulated into the solid lipid matrix and released in a controlled manner. SLN are generally spherical in shape, with particle sizes of 10–1000 nm [52,53]. The use of physiological lipids in the formulation improves their biosafety and bio-efficacy. SLN combine the advantages of the traditional drug carriers, including lipid and polymeric nanoparticles, and overcome the limitations associated with them. SLN have shown the potential to improve drug stability and bioavailability via different routes of administration.

### 3.4. Nanostructured Lipid Carriers

Nanostructured lipid carriers (NLC) have emerged as a promising second generation of lipid nanoparticles, the first one being SLN [54,55]. This new unstructured-matrix SLN is made up of a mixture of blended solid and liquid lipids as a core matrix, and an aqueous phase containing a surfactant or a mixture of surfactants [54,56,57,58]. Usually, solid lipids are mixed with liquid lipids in various ratios from 70:30 to 99.9:0.1, respectively, while the surfactant content ranges between 1.5 and 5% (*w*/*v*) [56].

Due to the incorporation of biodegradable and compatible liquid lipids into the solid matrix, NLC were found to offer several advantages in drug delivery over SLN, in addition to the possibility of encapsulating both lipophilic and hydrophilic drugs. Other advantages include increased drug loading capacity and solubility, enhanced storage stability, improved permeability and bioavailability, as well as reduced adverse effects, prolonged half-life, and tissue-targeted delivery [56,59,60].

### 3.5. Emulsions

#### 3.5.1. Nano- and Microemulsions

Nano- and microemulsions are the most famous colloidal dispersions. They are colloidal systems composed of two immiscible liquids. Both of these systems comprise an aqueous phase, an oil, a surfactant, and a cosurfactant or cosolvent [59,60]. Their nanoparticles have some basic structures with hydrophobic and hydrophilic cores, which are arranged according to the external phase [61]. They are generally classified as water-in-oil and oil-in-water emulsions. They can be prepared through spontaneous emulsification or by high-energy methods, i.e., micro-fluidization or high-pressure homogenization [62,63].

Although they show many similarities, such as transparency, being isotropic, and low viscosity, they are naturally different. Nanoemulsions are conventional emulsions with particle size strictly <200 nm, while microemulsions are in fact “swollen micelle” systems in which the internal phase is incorporated into the core of surfactant micelles under specific environmental conditions and compositions [64,65,66]. Physiochemically, nanoemulsions are thermodynamically unstable systems, while microemulsions are thermodynamically stable systems [65]. The free energy of nanoemulsions is higher than that of the separate formulation components, while the free energy of the separate components of microemulsions is higher than that of the microemulsion. Unlike microemulsions, nanoemulsions are kinetically stable systems: they present high-energy barriers and a slow mass transport phenomenon, which slow down their destabilization process [67].

Nano- and microemulsions have been described as ideal carriers for the delivery of drugs via many administration routes, including oral, pulmonary, ocular, and parenteral [68,69,70].

#### 3.5.2. Self-Emulsifying Drug Delivery Systems

Although nano- and microemulsions are comparatively stable systems, certain factors such as poor palatability, drug hydrolysis, and long-term stability limit their use [62,63]. Issues related to nano- and microemulsions have been addressed by the introduction of self-(nano)emulsifying drug delivery systems (S(N)EDDS). They are defined as isotropic mixtures of oils, surfactants, and cosurfactants or cosolvents, which can spontaneously form a (nano)emulsion with droplet sizes of 200 nm or below upon contact with gastrointestinal fluids [71]. They consist of oils (<30% *w*/*w*) and high amounts of surfactants and cosurfactants (>60%). As they can be packed into capsules as a single dose and do not contain water, S(N)EDDS offer improved palatability, physical and chemical stability, and patient compliance. Specific advantages associated with S(N)EDDS include high drug loading, improved drug absorption, controlled delivery, targeting potential, and minimized variability resulting from food effects [46]. Not only can they improve drug bioavailability, but S(N)EDDS are made from excipients regarded as safe, and are easy to manufacture and scale up. S(N)EDDS are one of the technologies applied for the improved delivery of anti-TB drugs.

## 4. Trends in Lipid-Based Drug Delivery Research for TB

The formulation approaches investigated for improving the encapsulation and delivery strategies of drug molecules to bring about potential improvements in TB management to date are briefly illustrated. Table 1 summarizes the key components and particularities of the lipid-based formulations commonly investigated for TB therapy, with the reports being cited in chronological order of publication.

### 4.1. Liposomes

Numerous studies have been conducted to evaluate liposomes as vehicles for anti-TB drugs. In addition to enhancing the therapeutic response, liposomes have been reported to be the most suitable and safe pulmonary vehicles of various antimicrobial drugs [12,76]. This is firstly due to their general biocompatible nature, particularly because of their similarity with lung surfactants (being both made of phospholipids), which allows for the avoidance of any possible local irritation [12,76]. Apart from their top ranking as the most clinically established drug carriers [111], liposomes have gained tremendous consideration in targeting intramacrophage pathogens due to their well-known rapid uptake by macrophages [112], where *M. tuberculosis* often resides [15]. In addition, considerable efforts have been dedicated to exploiting liposomal encapsulation as part of the solubilization strategies to ensure improved pharmacokinetic profiles of anti-TB drugs or enhanced product biocompatibility and efficacy.

Liposomes loaded with rifampicin have been developed using the thin film hydration technique followed by the freeze-drying technique to produce inhalable liposomal particles [81]. The liposomal formulation containing soy lecithin and cholesterol in a 3:2 mass ratio and drug to lecithin in a 1:5 mass ratio was deemed to be the best based on an encapsulation efficiency of 79.25%. With a controlled and sustained release profile, the optimized liposomes demonstrated much better pharmacokinetic patterns than the free drug following intra-tracheal instillation and oral administration to Wister rat models [81]. However, no insight was provided into the antimycobacterial efficacy of the formulated liposomes to confirm the delivery of the encapsulated drug in biological media.

In another study, rifampicin-loaded inhalable liposomes were formulated by the thin film hydration method using either natural soy phosphatidylcholine or its hydrogenated derivative with cholesterol or oleic acid [78]. The addition of cholesterol was found to increase the particle size while the presence of oleic acid was found to decrease the viscosity of the inhalable formulation developed. The selected drug-loaded liposomes were those made of natural soy phosphatidylcholine and cholesterol in a mass ratio of 3:1. The optimized rifampicin-loaded liposomes exhibited much better cell uptake, with reduced cytotoxicity towards alveolar epithelial cells, than the liposomes containing oleic acid, which dramatically reduced cell viability [78]. Nonetheless, the antimycobacterial activity of the proposed formulation was not reported.

Patil-Gadhe et al. [79] formulated and evaluated the in vitro antimycobacterial activity and cytotoxicity of inhalable rifapentine-loaded liposomes [79]. With a rifapentine concentration of 10 g/mL, the prepared liposomal formulation showed high antimicrobial efficacy and no cytotoxicity in A549 cells [79]. In addition, the rifapentine-loaded proliposomes were found to be safe in Wistar rats at concentrations of 1–5 mg of rifapentine/kg. However, doses ≥ 10 mg/kg were fatal to the animals, indicating the need for optimization of the balance between the activity and biocompatibility of the developed liposomes [79].

To produce a dual therapeutic system for tuberculosis treatment, viz. pulmonary medication and lung surfactant mimicking action, inhalable isoniazid-loaded liposomes were formulated by the thin film hydration method using dipalmitoyl phosphatidylcholine (DPPC), the main component of lung surfactant [76]. These liposomes exhibited a good anti-mycobacterial response and relieved lung surfactant depletion in TB-infected animals. This was deemed to be useful for improving the pathological conditions related to the depletion of endogenous lung surfactant due to internal or external causes such as the presence of *M. tuberculosis* in alveoli. Moreover, the absence of cholesterol as a lipid helper was justified by its possible inhibitory effect on the potential of DPPC to act as lung surfactant [76].

Another illustrative example of the excellent pulmonary biocompatibility of liposomes includes the development and commercialization of phospholipid mixtures as artificial lung surfactants, with branded names such as Survanta^®^ for the prophylaxis treatment of respiratory distress syndrome in neonates [113]. The use of such biocompatible lipid materials for the development of liposomes targeting pulmonary tuberculosis would lead to an attractive breakthrough with easy clinical translation. However, further efforts are needed to co-load several anti-TB drugs in such liposomes to ensure perfect matches with the existing multidrug TB regimen, which is recommended for tackling the issue of drug resistance [2].

Previous research in liposome engineering has also addressed the concepts of the targeted and sustained release of anti-TB drugs in the area of interest. Pandey et al. [74] developed multilamellar liposomal vesicles co-loaded with rifampicin and isoniazid using phosphatidylcholine and cholesterol in a 2:1.5 mass ratio. Through in vivo studies, it was observed that there was remarkable sustained drug release in alveolar macrophages following pulmonary administration to guinea pigs. In addition, the pure drugs could not last over one day in the plasma. Therefore, these findings showed that a single nebulization of the prepared liposomal formulation offered an effective plasmatic drug concentration that was stable for up to two days. The encapsulated drugs were recovered in the lungs five days from initial nebulization; however, their antimycobacterial activities were not reported.

The use of liposomes for targeting lung alveolar macrophages as part of a lung-macrophage-targeting strategy for TB has been demonstrated [73]. In this study, the authors formulated inhalable rifampicin-loaded liposomes using egg phosphatidylcholine and cholesterol in a 7:3 mass ratio. On one hand, these liposomes were decorated with maleylated bovine serum albumin and O-steroyl amylopectin as macrophage-receptor-targeting ligands and, on the other hand, dicetyl phosphate was incorporated as a negatively charged agent. The ligand-anchored and negatively charged liposomal aerosols demonstrated enhanced drug concentrations in alveolar macrophages over a prolonged period. In addition, these liposomes exhibited a much higher clearance rate of *M. smegmatis* residing in rat macrophages (89–93%) than the pure drug and non-decorated neutral liposomes (54.3% and 68.4%, respectively). This explains the preferential accumulation of the surface-modified liposomes in the macrophages over the conventional liposomes and the free drug. Nonetheless, since the developed formulation was a mono-drug system, extensive studies are required to add on other anti-TB drugs and evaluate the in vivo performance of the resultant liposomes for multidrug therapy.

In addition to achieving targeted drug delivery, Kaul et al. [114] combined the ligand surface decoration approach with the use of imaging agents to monitor liposome localization and fate in biological systems. These authors developed folate-receptor-targeted liposomes labeled with technetium (^99^mTc) for bio-imaging and the non-invasive co-delivery of rifampicin and ofloxacin to activated macrophages. The tissue distribution studies revealed high blood pool activity of up to 24 h post injection, with preferred localization in the spleen, liver, and kidney one hour after injection. The therapeutic efficacy of the formulation in the murine model of infection was confirmed, but no toxicological data were reported.

The macrophage-targeting approach is a reputed concept in anti-TB drug delivery, but the intracellular fate of the liposomal cargoes raises concerns in conjunction with the expected biological activity. In fact, following the macrophage uptake via phagocytosis, liposomes are delivered to phagolysosomes, where their components are subjected to extensive enzymatic digestion and/or acidic degradation, which can lead to the reduction or loss of biological activity [115]. Since a gradual decrease in pH occurs in the phagocytotic pathways, from early phagosomes (pH 6.5) to phagolysosomes (pH 4.5), the use of acid-triggered release from liposomes has been introduced as a potential strategy to prevent cargo digestion during the phagocytotic process.

pH-sensitive liposomes are designed to ensure cargo release upon macrophage uptake in early phagosomes prior to reaching the phagolysosomes. This allows the encapsulated drug to diffuse into the cytosol earlier to display the expected biological action and escape from further potential deactivation [115,116]. To this effect, the use of pH change as a stimulus for controlled release has been applied to enhance the intra-macrophage delivery and minimize the premature “off-target” release of anti-TB drugs.

Bhardwaj et al. [82] developed isoniazid- and ciprofloxacin-co-loaded liposomes containing 4-aminophenyl-α-D mannopyranoside as a macrophage-targeting agent. Cholesteryl hemi-succinate (CHEMS) and 1,2-dioleoyl-sn-glycero-3-phosphoethanolamine (DOPE) were used for liposome preparation as pH-sensitive agents. The formulated pH-sensitive ligand-decorated liposomes exhibited much higher alveolar macrophage uptake than the non-decorated liposomes. Following pulmonary delivery, these liposomes achieved the maximum concentration of the drugs in the lungs. The results of this study are noteworthy evidence for the possibility of improving the bioavailability of anti-TB drugs by means of pH-sensitive macrophage-targeting liposomes.

However, while liposomes are promising as reputable vehicles for anti-TB drugs, the cost of the existing liposomal technology remains a hindrance. The costly status of liposome formulation arises mostly from the use of sophisticated equipment, such as microfluidic systems [37], and expensive materials [112,117]—especially for the preparation of pH-sensitive liposomes, where special lipids such as DOPE and CHEMS are commonly required [37,112].

To circumvent this, Nkanga et al. introduced crude soybean lecithin, a relatively cheap and readily available naturally occurring lipid mixture, as a cost-effective lipid for the affordable liposomal encapsulation of anti-TB drugs [85]. The preliminary studies demonstrated much better liposomal encapsulation of isoniazid (INH) for the crude soybean lecithin than the purified soybean lecithin. Nonetheless, the release study performed at pH 7.4 revealed a sudden release feature, indicating the potential premature burst release that can lead to the “off-target” loss of INH before reaching the infected site. The need for improving the liposomal encapsulation and delivery of INH as a small hydrophilic drug has therefore become a topic of current interest.

To address the issue of liposome leakage from the crude soybean lecithin liposomes, Nkanga et al. further investigated the liposomal encapsulation of a hydrazone derivative of isoniazid, namely isonicotinic acid (4-hydroxy-benzylidene)-hydrazide, for pH-dependent controlled release [87]. The mentioned hydrazone derivative was encapsulated in liposomes using crude soybean lecithin for potential cost-effective developments. The INH-HB loaded liposomes exhibited remarkable pH-responsive INH release profiles that reached 100% at pH 4.4, compared to 22% at neutral pH (7.4) [87].

From this experimental trial, Nkanga and Krause [118] undertook an extended study by conjugating INH to a hydrophobic fluorescent tag, zinc phthalocyanine, through hydrazone linkages for the development of a multifunctional liposomal tool for anti-TB theranostic applications (combining drug delivery with fluorescence bioimaging). The INH-grafted-phthalocyanine hydrazone derivative (PC-INH) was loaded into liposomes by the thin film hydration method using crude soybean lecithin to maintain low costs. The prepared liposomes demonstrated distinctive pH-triggered INH release behavior in vitro, confirming the potential of hydrazone–drug conjugate liposomes to achieve controlled drug release. In addition to pH-sensitive release characteristics, the formulated liposomes showed good visible light absorption, which makes this a promising multifunctional system for multimodal applications. However, the biological activities of the synthesized hydrazone–drug conjugates and their liposomal formulations were not investigated.

Miretti et al. recently reported the good photodynamic activity of liposomes containing zinc phthalocyanine against both drug-susceptible and multidrug-resistant strains of *M. tuberculosis*, which could be encouraging for further evaluation of PC-INH for synergetic photodynamic antimycobacterial chemotherapy [88].

In another study, Nkanga and Krause [9] addressed the need for the imperative use of organic solvents for the liposomal encapsulation of large hydrophobic compounds by means of crude soybean lecithin. Subsequently, using PC-INH as a drug model due to its hydrophobic nature, the potential of cyclodextrin complexation was demonstrated as a strategic pre-treatment for the organic, solvent-free liposomal encapsulation of hydrophobic compounds. The complex-loaded liposomes met the critical quality attributes, viz. particle size, zeta potential, particle size distribution, and acceptable shelf stability, as well as remarkable pH-dependent INH release profiles set out at the start of the experiments. In addition, the prepared inclusion complexes showed some fluorescent behavior deemed to be useful for image-guided drug delivery studies [9].

However, due to the mono-drug composition of the reported complex-loaded liposomes, Nkanga et al. set out to co-encapsulate the previously reported complex with rifampicin as a second TB drug [90]. The aim of this study was to extend the therapeutic value of the developed crude soybean lecithin liposomes to fulfil the multidrug therapy concepts of TB management, thus avoiding a potential rise in drug resistance. Nkanga et al. successfully co-loaded rifampicin, the complex of cyclodextrin, and PC-INH in crude soybean lecithin liposomes under organic, solvent-free conditions using a heating method [90]. The formulated dual liposomes demonstrated pH-dependent, controlled co-delivery for the two anti-TB drugs, good biocompatibility, and marked penetration through the biological membranes of human lung fibroblasts and epithelial cells. Although the study provides preliminary insights into the biological performance of crude soybean lecithin liposomes, the complexity of this liposomal system is in question, as far as the overall drug load is concerned. Furthermore, extensive pharmacological studies are needed to evaluate the bioavailability and antimycobacterial efficacy of the encapsulated drug molecules in vivo.

### 4.2. Niosomes

Being part of the bilayered lipidic systems, niosomes have been mainly used as nano-carriers for controlling the biodistribution and improving the pharmacokinetics of anti-TB drugs. In a study conducted by Jain et al., it was demonstrated that rifampicin-loaded niosomes can be prepared with Span^®^ 85 and cholesterol in various molar ratios [91]. The in vivo distribution studies conducted in albino rats revealed that approximately 65% of the encapsulated rifampicin was localized in the lungs following injection through the caudal vein. This study focused on the biodistribution of the nanodrug, with no insights into the actual antitubercular activity or toxicity of the nano-formulations.

In a separate study, isoniazid-loaded niosomal delivery systems were investigated by means of biodistribution studies and controlled release possibility [94]. The niosomes were prepared using the reverse phase evaporation method. An extended and sustained drug release profile was observed, with a steady drug concentration occurring over 30 h. In addition, the drug-loaded niosomes exhibited high cell penetration (~62%) when tested on macrophages, which is an attractive feature for targeting mycobacteria. However, the antimycobacterial activity of the formulation developed was not discussed.

Similarly, Karki et al. [92] prepared isoniazid-loaded niosomes for controlled delivery. The in vitro release studies indicated that the total drug content was released within 48 h. In addition, a drug deposition study in healthy albino rats proved that the accumulation of the nano-drug in the visceral organs (lung, kidney, spleen) was much lower compared to that of the free drug. These in vivo data suggest that the developed niosomes are promising for avoiding systemic adverse effects. Nevertheless, the deposition rate of the formulation in the lung tissues was found to be quite low, which is disadvantageous for pulmonary TB that is mainly located in the lungs. Moreover, the biological activity and cellular uptake for the prepared niosomes were not reported.

The research into niosome technology for TB also investigated the impact of surface characteristics on the performance of the niosomal particles. El-Ridy et al. [93] investigated the loading of pyrazinamide in niosomes made of either Span^®^ 60 or 85 and cholesterol in different molar ratios. Dicetyl phosphate (DCP) and stearyl amine were used for impacting the particles with negative and positive surface charges, respectively. The highest entrapment was obtained for the formulation made of Span^®^ 60 and cholesterol in a molar ratio of 4:2. The negatively charged niosomes showed the highest encapsulation efficiency, followed by neutral niosomes. By conducting in vivo biological testing on guinea pigs infected with *M. tuberculosis*, it was observed that there was enhanced efficacy of pyrazinamide due to encapsulation in niosomes.

However, since the formulation only constituted one first-line TB drug, further studies are needed to co-load other anti-TB agents to ensure combination therapy prior to clinical product development. In fact, for anti-TB drug regimens comprising drug molecules of different solubilities, a thorough preliminary study is highly desired to ensure the successful co-loading of all the necessary drug molecules early on, before late-phase product development. Indeed, poor encapsulation of some of the small anti-TB drug molecules in niosomes has been previously reported. El-Ridy et al. [97] attempted to improve ethambutol hydrochloride’s activity and safety through encapsulation in niosomes. It was observed that the encapsulation of the drug in niosomes enhanced the lung targeting and biological activity over the free drug in guinea pigs. However, the authors observed poor encapsulation efficiencies (12–26%), which might not be ideal for clinical application.

A further study by Mehta et al. [95] investigated the possibility of the efficient co-encapsulation of anti-TB drugs in niosomes. The authors prepared highly stable niosomes using Triton X 100, PEG 2000, water, and Span^®^ 80 for the encapsulation of different first-line antitubercular drugs (rifampicin, isoniazid, and pyrazinamide). High encapsulation efficiency for each individual drug was observed, along with good system compatibility and stability. Nonetheless, there was no insight into the biological performance of the developed multidrug-loaded niosomes. Similar to this work, Mehta et al. [96] studied the encapsulation of rifampicin, isoniazid, and pyrazinamide into niosomes using tyloxapol as a biocompatible surfactant. It was observed that there was high encapsulation efficiency for each drug, as well as good stability of the resultant formulations. The localization study unveiled that isoniazid and rifampicin were likely to be present in the film bilayer, whereas pyrazinamide was adsorbed mainly on the surface of head groups. Sustained drug release was observed for isoniazid and pyrazinamide, while relatively faster release was observed for rifampicin, which can be attributed to the discrepancy in the solubilities of these drugs. At this stage, the elucidation of the in vivo activity of such multidrug-loaded niosomes would offer opportunities towards the development of niosomes matching the current multidrug regimens for anti-TB therapy.

### 4.3. Solid Lipid Particles

The trend in research for the development of SLN has included the design and evaluation of inhalable solid lipid nano- and micro-particles for enhancing the solubility, bioavailability, and efficacy of anti-TB drugs. The studies conducted in this field embrace the preliminary trials for formulation, optimization, and development as well as advanced pharmacokinetic and antimycobacterial assessment. A good example of a formulation design and development study is the work by Maretti et al. [101]. The work investigated the influence of pre-freezing conditions on the deep lung deposition of the inhalable formulation of rifampicin-loaded SLN [101]. The SLN were composed of stearic acid and sodium taurocholate. Variables such as the concentration of cryoprotectant, pre-freezing temperature, and SLN concentration in the formulation were studied at different levels. The most suitable conditions that showed a positive impact on the lung deposition of the prepared powder encompassed quick-freezing combined with certain levels of sample dilution, prior to the pre-freezing step without cryoprotectant. Though the thermal stability of rifampicin throughout the formulation process was assessed, system stability studies for the formulated inhalable dry powder were not reported. Another illustrative example of formulation development is the recent work reported by Nemati et al. [104], where the pulmonary administration of ethambutol encapsulated in SLN was investigated using dry powder inhalation. The prepared ethambutol-loaded SLN were composed of Compritol^®^ and Tween^®^ 80, which exhibited quite high drug encapsulation efficiency (98%). The MTT tetrazolium salt assay revealed the good biocompatibility and non-toxicity of the proposed formulations. Nonetheless, no antimycobacterial activity assessment was performed to verify the impact of the potential of the prepared SLN to release enough ethambutol molecules to ensure good mycobacterial clearance [104].

The design of solid lipid particles also involves the optimization of the system composition to confer the specific functionalities of the carriers, such as ionic charges or receptor-specific ligands on the surface for targeted delivery. As an example of the impact of surface charges, rifampicin chitosan-coated SLN represent a good illustration [103]. In this study, successful coating of spherical SLN composed of cetyl palmitate was confirmed by means of Fourier-transform infrared spectroscopy (FTIR) and surface charge analysis. The surface charge was assessed and found to vary from negative (–30 mV) for uncoated to positive (+40 mV) for chitosan-coated SLN (C-SLN). The C-SLN demonstrated much higher in vitro mucoadhesive properties and higher permeability in alveolar epithelial A549 cells than the uncoated SLN. The particle size of all the prepared SLN was found to be suitable for macrophages’ internalization, while the charge variation from negative to positive might have adversely affected this macrophage internalization and impacted the biological activity, since negatively charged particles are known to improve immune cells’ internalization [119]. Hence, macrophage uptake and antitubercular activity studies are highly desired to provide insights into the potential therapeutic performance of the developed C-SLN. Indeed, macrophage uptake experiments are presently established in the preliminary protocols for the assessment of potential anti-TB nanoparticles. To illustrate this fact, Gaspar et al. [102] investigated the potential of rifabutin-loaded SLN for pulmonary TB treatment. Two different formulations of SLN were produced, using glyceryl dibehenate and glyceryl tristearate as lipid constituents. These two formulations showed nanoparticle uptake of approximately 46% and 26%, respectively, using in vitro studies with THP1 cells differentiated in macrophages. In addition, the prepared SLN exhibited low cytotoxicity in lung cell lines and the freeze-dried formulations were found to be stable at 5 °C storage conditions. However, the antimycobacterial activity of the rifabutin-loaded SLN was not investigated, but this could offer more insights into the potential of the developed formulations for pulmonary TB therapy.

Similarly, Maretti et al. demonstrated macrophage targeting based on surface charge. They evaluated inhalable SLN for the deep lung delivery of rifampicin [100]. These lipid nanocarriers were composed of stearic acid and sodium taurocholate and exhibited a suitable aerodynamic diameter for delivery to the alveolar epithelium. The negatively charged particles showed improved uptake by the macrophages, as well as preserving their antibacterial activity when tested on *Bacillus subtilis* strains. However, the formulation design and evaluation of the actual inhalable products for this promising delivery system were not investigated. In addition to surface charge decoration, the use of specific ligands to decorate the solid lipid particles’ surfaces and target the site of interest for tuberculosis has been also explored [57].

With targeted delivery not being sufficient to enhance antimicrobial activity, further research in solid lipid particles has focused on controlling and sustaining the release of the encapsulated drug. Aboutaleb et al. [99] observed improved antitubercular activity of rifampin following its encapsulation in SLN using cetyl palmitate and Tween^®^ 80/Poloxamer 188 [99]. The formulation exhibited sustained release of rifampin for 72 h, followed by enhanced antimycobacterial efficacy. However, the prepared SLN showed less dense negative surface charges, which could adversely impact the stability profile of the formulation. In addition, the proposed formulation being a mono-drug system, its clinical application may not be approved, since the use of monotherapy for TB is no longer recommended due to the alarming emergence of resistant strains of *M. tuberculosis* [11].

In the context of multidrug therapy, an excellent attempt in solid lipid particle research for TB was demonstrated in a study by Pandey et al. [98]. The study evaluated the chemotherapeutic potential of nebulized solid lipid particles containing three different antitubercular drugs (rifampicin, isoniazid, and pyrazinamide). These solid lipid particles were made of stearic acid and prepared using the emulsion solvent diffusion technique. Due to good drug encapsulation, the residence time and drug bioavailability were considerably improved in specific organs (lung, liver, and spleen). The nebulized drug-loaded solid lipid particles exhibited much greater activity in *M. tuberculosis*-infected guinea pigs compared to the orally administered free drugs. No tubercle bacilli were observed in the lung/spleen after 7 days of treatment in guinea pigs with the proposed formulation. In addition, the biochemical investigation showed no hepatotoxicity following the administration of the prepared solid lipid particles. Nevertheless, basic studies elucidating the solid lipid particles’ uptake by macrophages are needed to ensure the effective treatment of latent tuberculosis, since the mycobacterium is often hidden in the granuloma [120].

### 4.4. Nanostructured Lipid Carriers

Several studies have demonstrated the potential of NLC as a drug vehicle for TB management. For instance, Song et al. [105] designed mannosylated NLC for the targeted delivery of rifampicin to alveolar macrophages. The diameter size of the developed NLC (160 nm) was deemed to be acceptable for deep lung deposition [105]. Rifampicin-loaded NLC led to much greater drug accumulation in the lungs than a rifampicin solution. Moreover, rifampicin-loaded mannosylated NLC exhibited significantly higher uptake efficiency in cells and alveolar macrophages than rifampicin-loaded NLC without mannose, which underlines the targeting capabilities of the proposed formulations [105]. Obtained data indicated that mannosylated rifampicin-loaded NLC decreased the intracellular growth of *M. avium* infecting bone-marrow-derived macrophages [57].

In another study, Pinheiro et al. formulated mannosylated NLC encapsulating rifabutin [121]. A faster drug release profile (~50% after 5 h) was observed at pH 5.0, while only ~20% of the drug was released at pH 6.2 and 7.4. After 25 h of the release experiment, rifabutin release reached 100% at pH 5.0, while < 40% release was observed at neutral pH. Based on these results, the developed NLC were proposed for pH-dependent delivery inside the phagolysosomes (pH ~ 5.0), where the etiologic agent of TB is located. Nonetheless, no insights into pharmacokinetics and pharmacodynamic profiles were provided.

Similarly, Carneiro et al. developed rifampicin-laden NLC that were functionalized with a tuftsin-modified peptide [122]. Following 24 h of incubation, targeted NLC were found to be more significantly internalized by alveolar macrophages than NLC without peptide. Furthermore, both NLC were two-fold more effective in vitro against *M. tuberculosis* than free rifampicin [122]. In contrast, the unloaded NLC did not affect the mycobacterium growth. These results evidenced the ability of tuftsin-modified peptide to selectively recognize receptors located on infected alveolar macrophages, thereby enhancing the cell uptake of the nanocarriers and subsequent intracellular cargo delivery [122]. However, the antimycobacterial activities of these formulations were not reported.

In addition to conventional ligand-decorated NLC, several research groups have explored polymer-coated NLC for anti-TB drug delivery. For example, NLC modified with a chitosan–acemannan conjugate were used as a drug delivery system for the intracellular targeting of rifampicin in vitro. Data indicated excellent cell internalization [123]; however, the in vivo therapeutic efficacy of these carriers was not reported. A similar formulation is polysorbate-coated sophorolipid-based NLC, which were developed for the targeted delivery of rifampicin [124]. With sustained drug release of 22.3% after 5 h, the developed NLC were deemed to be greatly suitable for the long-term management of TB. In addition, the lactonic sophorolipid being a biosurfactant with reputed antimicrobial activity, the proposed formulation appears to be promising for the management of TB. However, no anti-TB efficacy assessments have been done to verify this hypothesis.

An attractive characteristic of NLC technology that makes it an intriguing delivery platform for anti-TB therapy is the ability to co-load multiple therapeutic agents of different solubilities. Banerjee et al. engineered NLC loaded with rifampicin and isoniazid [125]. These dual-drug-loaded NLC achieved phago-lysosomal trafficking; data indicated that these carriers effectively co-localized with different cellular compartments (lysosome and endosome) of macrophages [125]. Following oral administration, the findings revealed that the formulated NLC enhanced the pharmacokinetic profile of different drugs and overall increased their relative bioavailability by many folds when compared to unformulated drugs.

Certainly, the possibility for oral drug delivery has been identified as one of the key advantages of NLC. These lipid carriers have shown the potential to facilitate in vivo anti-TB applications of copper (II) complexes, which show excellent antimicrobial activity in vitro but no efficacy in vivo, owing to their poor oral bioavailability, related to their low aqueous solubility [126]. The encapsulation of copper (II) in NLC led to greater anti-TB activity, associated with much lower in vivo acute toxicity than the free copper (II) complexes [127,128]. As an emerging platform technology, the NLC appear to be promising for TB management; however, further research efforts are required to boost their translational development to fully unfold among advanced nano-formulations.

### 4.5. Emulsions

Research in the area of therapeutic emulsions has addressed fundamental questions related to the structure stability of emulsions on the incorporation of anti-TB drugs. Mehta et al. [107] formulated a microemulsion as an advanced delivery system for rifampicin. This microemulsion was composed of oleic acid, Tween^®^ 80, ethanol, and phosphate buffer. The authors observed that the incorporation of rifampicin induced structural changes, from a water-in-oil microemulsion into an oil-in-water microemulsion that remained stable at infinite dilution. The dissolution studies revealed the controlled release of rifampicin from the oil-in-water emulsion droplets, which was encouraging for further investigations. In a subsequent study, Mehta et al. [129] incorporated a second anti-TB drug (isoniazid) into the same Tween^®^ 80-based microemulsion. The physicochemical analysis unveiled major changes in the structure of the emulsions upon the dissolution of rifampicin, which caused system conversion from water-in-oil to oil-in-water. Interestingly, the resultant oil-in-water microemulsion remained stable on incorporation of the hydrophilic drug, isoniazid. Spectroscopic studies demonstrated that isoniazid molecules were present in the continuum region of the oil-in-water microemulsion. However, although the latter formulation was a dual system (with rifampicin and isoniazid), the authors did not report bioassay data discussing the potential effectiveness of the prepared microemulsion for the treatment of tuberculosis.

Further basic research focused on extending the therapeutic values of microemulsions by attempting to co-load some more first-line anti-TB drugs to meet the currently recommended multidrug regimens. Kaur et al. [108] carried out structural investigations of microemulsions encapsulating three anti-TB drugs of various solubilities, including rifampicin, isoniazid, and pyrazinamide. The authors attempted to monitor the change in the microstructure of non-ionic Brij^®^ 96 microemulsions and to locate the solubilization loci of these antitubercular drugs. By monitoring the spectral shift, Stroke’s shift, and interactions with anisotropic dyes, the authors observed that rifampicin and isoniazid were both located at the interface, but towards the oil body and hydrophilic side, respectively. Pyrazinamide was found to remain in the dispersing medium (free water). Differential scanning calorimetry also confirmed the stability and location of the drugs in the Brij^®^ 96 microemulsions. The states of water in the micro-heterogeneous environment and their variation, viz. free, bound, interphasal, and non-freezable water, upon dilution were elucidated. Nonetheless, there were no insights into the antimycobacterial activity of the encapsulated drug molecules for optimal anti-TB multidrug therapy.

Subsequently, Kaur et al. [109] again investigated the co-encapsulation of isoniazid, pyrazinamide, and rifampicin in Brij^®^ 96 microemulsions. In the co-loaded microemulsions, the antitubercular drugs were found to occupy the same solubilization sites as in the case of single-drug-loaded microemulsions. In vitro release studies indicated that isoniazid and pyrazinamide exhibited a diffusional Fickian release mechanism, whereas rifampicin exhibited an anomalous release mode. The cytotoxicity of the formulation was proven to be dependent on the concentration and colloidal structure of the microemulsion. Nevertheless, no biological studies were conducted to verify whether there were improvements in the antitubercular activity and stability of the anti-TB drugs following encapsulation into the microemulsions. Hussain et al. encapsulated rifampicin in a solid SNEDDS using Capmul^®^ MCM C8 as oil, Labrasol^®^, and Cremophor-EL. The Labrasol^®^-assisted rifampicin solubilization caused the immediate release of the drug [110] at different pH values. In vivo permeation and biopsy studies revealed improvements in the intestinal permeation of rifampicin. The authors observed good in vivo–in-vitro correlation, with predicted systemic absorption of approximately 96%. The rifampicin-loaded solid SNEDDS were found to have better pharmacokinetic profiles than the rifampicin suspension, which makes this a good alternative to conventional delivery systems for TB management. However, further investigations are required to extend the therapeutic value of the developed SNEDDS, by co-loading other anti-TB drugs, thus eliminating drug resistance concerns, in order to undertake antimycobacterial bioassays and evaluate the potential therapeutic efficacy of the proposed formulations.

## 5. Conclusions

Tuberculosis (TB) remains a leading cause of death worldwide, and its treatment is challenging due to the poor pharmacokinetic profiles of existing TB drugs. In addition, the pathogen is highly infectious and very resilient to strong acids and bases because of its cell wall, which comprises approximately 40–60% complex lipids. Consequently, targeted therapy based on understanding the structure and biosynthesis of the bacterium is cardinal in effectively managing the disease as well as eradicating drug resistance. The current and traditional drug delivery systems require high-dose-frequency administration, which impacts negatively on patient adherence and consequently leads to treatment failure and the development of multidrug-resistant strains. Novel approaches such as the use of liposomes, niosomes, solid lipid nanoparticles, nanostructured lipid carriers, nano- and microemulsions, and self-emulsifying formulations have shown significant potential to improve the bioavailability, stability, and safety of TB drugs. The factors that influence the performance of targeted pulmonary delivery systems using lipid-based delivery systems have been highlighted. Overall, lipid-based systems have shown great promise in anti-TB drug delivery applications. While this underscores the need to fast-track the translational development of these drug delivery systems to tackle the deadly reputation of TB, it is important to note that there are few lipid-based nanomedicines available on the market due to scalability concerns, the overall cost to patients, and the need for highly specialized equipment. Further work may be required to be able to translate this valuable technology from bench to pharmaceutical market.

## Figures and Tables

**Table 1:** Brief presentation of lipid-based formulations investigated for encapsulation and delivery of anti-TB drugs.

Vehicles	Composition	Drug Molecules	System Specificity and Functionality	Reference
**Liposomes**	Phosphatidylcholine, cholesterol	Isoniazid, pyrazinamide, rifampicin, ethionamide, streptomycin	Attempt for multiple drug encapsulation in liposomes; co-encapsulation of isoniazid and pyrazinamide was successful whereas rifampicin, ethionamide, and streptomycin was not substantial	[72]
	Egg phosphatidylcholine, cholesterol, maleylated bovine serum albumin, O-steroyl amylopectin/dicetylphosphate	Rifampicin	Enhanced drug concentration in alveolar macrophages, a higher clearance rate of *M. smegmatis* in the rat macrophage, improved efficiency with aerosol formulation	[73]
	Phosphatidylcholine, cholesterol	Rifampicin Isoniazid	Sustained drug release in alveolar macrophages by pulmonary administration to guinea pigs	[74]
	Egg yolk phosphatidylcholine type XI-E, dipalmitoylphosphatidylcholine, cholesterol	Rifampicin Isoniazid	Co-loading increased the encapsulation and extended the release of both drugs	[75]
	Dipalmitoyl phosphatidylcholine (DPPC)	Isoniazid	Deep lung deposition (27%), effective delivery of isoniazid, lung surfactant mimic action	[76]
	Soybean phosphatidylcholine, cholesterol, mannitol	Isoniazid	Proliposomes with attractive flowability, powder performance, and promising biological effect	[77]
	Soy phosphatidylcholine/hydrogenated derivative, cholesterol, oleic acid	Rifampicin	Good cellular uptake and less toxicity towards alveolar epithelium for the formulation without oleic acid	[78]
	Hydrogenated soy phosphatidylcholine, cholesterol	Rifampetine	Antimicrobial efficacy without cytotoxicity in A549 cells	[79]
	Phospholipid (Lipoid S-75), sulfphobutyl ether P-cyclodextrin, vitamin C	Rifampicin	Good flowability, aerodynamic diameter for pulmonary delivery, good in vitro antitubercular activity	[80]
	Soy lecithin, cholesterol	Rifampicin	Controlled and sustained release behavior, better pharmacokinetic profile	[81]
	4-aminophenyl-a-D mannopyranoside as a macrophage-targeting agent. Cholesteryl hemisuccinate (CHEMS) and 1,2-dioleoyl-sn-glycero-3-phosphoethanolamine (DOPE)	Isoniazid Ciprofloxacin	pH stimuli release optimal at macrophage acidic conditions, high alveolar macrophage uptake, and pulmonary delivery of drug achieved	[82]
	Hydrogenated phosphatidylcholine from soybean, cholesterol, α-tocopherol, and folate-MPEG2000-DSPE	Rifampicin Ofloxacin	Efficient antimicrobial activity in vitro and in murine models, enhanced pharmacokinetic profiles, macrophage-targeting activity, and particulates endowed with radiolabeling properties for visualization	[83]
	D-erythro-sphingosine-1-phosphate (S1P); lysobisphosphatidic acid (LBPA) or arachidonic acid, L-α-phosphatidylserine	Phosphatidic acid Phosphatidylinositol 3-phosphate Phosphatidylinositol 5-phosphate	Increased intracellular death of *Mycobacteria* BCG and *Pseudomonas aeruginosa* by phagosome acidification and ROS generation	[84]
	Crude soybean lecithin and cholesterol	Isoniazid	Crude soybean lecithin liposomes exhibited much higher encapsulation efficiency for isoniazid than purified soybean lecithin liposomes, introducing the crude product for cost-effective drug encapsulation	[85]
	Dimethyldioctadecylammonium (DDA), monophosphoryl lipid A (MPLA), trehalose 6,6′-dibehenate (TDB)	DNA vaccine	Slow and prolonged release of DNA, enhanced and persistent protection against TB, increased storage stability of the vaccine	[86]
	Crude soybean lecithin and cholesterol	Isonicotinic acid (4-hydroxy-benzylidene)-hydrazide	Crude soybean lecithin liposomes showed high encapsulation efficiency for hydrazone–drug conjugates and controlled release of isoniazid at different pH	[87]
	Crude soybean lecithin	Isoniazid-grafted zinc phthalocyanine	The conjugation of chemotherapeutics to phthalocyanines as a potential strategy for liposomal controlled release was successfully established	[87]
	Dipalmitoylphosphatidylcholine, cholesterol	Zinc phthalocyanine	Inactivation of sensible and multidrug-resistant strains of *M. tuberculosis* by photodynamic activity	[88]
	Crude soybean lecithin	Inclusion complexes of cyclodextrin with isoniazid-grafted zinc phthalocyanine	The use of cyclodextrin complexation to facilitate liposomal encapsulation of hydrophobic compounds under organic, solvent-free conditions was introduced	[9]
	Crude soybean lecithin	Rifampicin Isoniazid	The feasibility of using crude soybean lecithin for preparation of combination products for liposomal dual delivery was demonstrated	[89]
	Crude soybean lecithin	Rifampicin and isoniazid-grafted zinc phthalocyanine	The prepared liposomes demonstrated pH-dependent controlled dual delivery of the two drugs, good biocompatibility, and marked uptake by the lung fibroblasts and epithelial cells	[90]
**Niosomes**	Span^®^ 85, cholesterol	Rifampicin	Good distribution with lung affinity of approximately 65% due to the controlled size of particles	[91]
	Span^®^ 60, cholesterol	Isoniazid	Low accumulation of drugs in visceral organs (lung, kidney, spleen)	[92]
	Span^®^ 60/85, cholesterol, dicetyl phosphate/stearyl amine	Pyrazinamide	Improved drug efficacy in guinea pigs infected with *M. tuberculosis*	[93]
	Span^®^ 20/60, cholesterol, di-cetylphosphate	Isoniazid	Prolonged delivery in treated sites and high macrophage uptake of negatively charged particles	[94]
	Triton X 100, polyethylene glycol (PEG) 2000, Span^®^ 80	Rifampicin Isoniazid Pyrazinamide	Stability and compatibility of drugs in niosomes, release of rifampicin and isoniazid by a Fickian mechanism, and a non-Fickian release observed for pyrazinamide	[95]
	Tyloxapol, PEG 2000	Rifampicin Isoniazid Pyrazinamide	Stability of the formulation, isoniazid released by a Fickian diffusion, rifampicin and pyrazinamide by a non-Fickian mechanism	[96]
	Span^®^ 60/85, cholesterol, dicetyl phosphate/stearyl amine	Ethambutol	Good stability for neutral and positively charged niosomes	[97]
**Solid lipid nanoparticles**	Stearic acid	Rifampicin Isoniazid Pyrazinamide	Good aerodynamic size for broncho-alveolar delivery, bioavailability, greater activity in *M. tuberculosis* infected guinea pigs, and no hepatotoxicity induced	[98]
	Cetyl palmitate, Tween^®^ 80/Poloxamer 188	Rifampin	Improved antitubercular activity and sustained release of rifampin	[99]
	Stearic acid, sodium taurocholate	Rifampicin	Appropriate aerodynamic size for pulmonary delivery to alveolar epithelium, with good respirability fraction (>50%) and activity against *Bacillus subtilis* strains	[100,101]
	Glyceryl dibehenate/glyceryl tristearate, Tween^®^ 80	Rifabutin	The macrophage uptake of 46% for nanoparticles made with glyceryl dibehenate and low cytotoxicity effect on lung cell lines	[102]
	Cetyl palmitate, chitosan	Rifampicin	Higher in vitro mucoadhesive properties and permeability in alveolar epithelial cells	[103]
	Comptitol, Tween ^®^ 80	Ethambutol	Biocompatible, non-toxic particles, dry powder inhaler suitable for pulmonary delivery	[104]
**Nanostructured lipid carriers**	Polyoxyethylene 40 stearate, caprylic/capric triglyceride, and polyoxyl 40 hydrogenated castor oil, Poloxamer 407, cetyltrimethylammonium bromide	Rifampicin	Improved uptake of drug in alveolar macrophages	[105]
	Precirol^®^ATO 5, polysorbate 60, miglyol-812, mannose	Rifampicin	Efficient uptake by bone-marrow-derived macrophages and decrease in the intracellular growth of the mycobacteria	[57]
	Lipoid S-75, Tween 80, Poloxamer 188, Precirol^®^ ATO-5, glyceryl distearate, squalene	Rifampicin	Enhancement of pharmacokinetic parameters and improvement of drug bioavailability	[106]
**Emulsions**	Oleic acid, phosphate buffer, Tween 80, ethanol	Rifampicin	Controlled release of rifampicin achieved	[107]
	Oleic acid, phosphate buffer, Tween 80, ethanol	Isoniazid	Stable formulation, isoniazid release by a non-Fickian release mechanism	[107]
	Ethyl oleate, Brij 96, Butanol	Rifampicin Isoniazid Pyrazinamide	Isoniazid and rifampicin located at the interface toward oil side, pyrazinamide remained in free water Isoniazid and pyrazinamide released by Fickian mechanism and rifampicin exhibited anomalous release	[108,109]
	Capmul MCM C8, Labrasol, Cremophor-EL	Rifampicin	Intestinal permeation of rifampicin facilitated, improved pharmacokinetic profile	[110]

## Data Availability

Not applicable.
